# Cardiac PET/MRI—an update

**DOI:** 10.1186/s41824-018-0050-2

**Published:** 2019-01-22

**Authors:** C. Rischpler, S. G. Nekolla, G. Heusch, L. Umutlu, T. Rassaf, P. Heusch, K. Herrmann, F. Nensa

**Affiliations:** 1 Department of Nuclear Medicine, University Hospital Essen, University of Duisburg-Essen, Essen, Germany; 2 Nuklearmedizinische Klinik und Poliklinik, Klinikum rechts der Isar, Technische Universität München, Ismaninger Straße 22, 81675 Munich, Germany; 3 DZHK (Deutsches Zentrum für Herz-Kreislauf-Forschung e.V.) partner site Munich Heart alliance, Munich, Germany; 4 Institute for Pathophysiology, University Hospital Essen, University of Duisburg-Essen, Essen, Germany; 5 Department of Diagnostic and Interventional Radiology and Neuroradiology, University Hospital Essen, University of Duisburg-Essen, Essen, Germany; 6 Department of Cardiology and Vascular Medicine, University Hospital Essen, West German Heart and Vascular Center, University of Duisburg-Essen, Essen, Germany; 7 Department of Diagnostic and Interventional Radiology, Medical Faculty, University Düsseldorf, Düsseldorf, Germany

**Keywords:** PET/MRI, Cardiovascular, Clinical applications

## Abstract

It is now about 8 years since the first whole-body integrated PET/MRI has been installed. First, reports on technical characteristics and system performance were published. Early after, reports on the first use of PET/MRI in oncological patients were released. Interestingly, the first article on the application in cardiology was a review article, which was published before the first original article was put out. Since then, researchers have gained a lot experience with the PET/MRI in various cardiovascular diseases and an increasing number on auspicious indications is appearing. In this review article, we give an overview on technical updates within these last years with potential impact on cardiac imaging and summarize those scenarios where PET/MRI plays a pivotal role in cardiovascular medicine.

## Introduction

In 2010, a new hybrid imaging device was installed for the first time for clinical use and raised great expectations: a truly integrated PET/MRI. Shortly thereafter, a first report on its technical characteristics and performance appeared (Delso et al. 2011). Further reports on its initial applications in oncological scenarios were released soon after. Of note, however, the first published manuscript on cardiovascular applications using this machine was a review article (Rischpler et al. 2013). Until the release of this review in 2013, no original article had been published and—not surprisingly—potential cardiovascular applications were only suggested, for a number of reasons: firstly, PET/MRI is a complex technology to which clinicians, physicists, researchers, and technologists had first to get used to. And this approach is obviously easier in organs as the brain that do not suffer from motion artifacts due to the heart beat and respiration. Secondly, patients with common cardiovascular applications in PET are still quite rare. Last but not least, and this is the experience of the authors, many cardiovascular imaging studies in PET/MRI are driven by specific, prospective research and not by clinical routine. As cardiac MRI has the advantage of a high soft tissue contrast and permits to assess functional parameters of the left and right ventricle with tissue characterization in great detail, PET has the potential of true molecular imaging with targets such as metabolic pathways, receptors, or cell surface markers. Until today, more and more promising applications of PET/MRI in cardiology have been reported and a guideline on hybrid cardiac PET/MRI has been published (Nensa et al. 2018a). The current review aims to give an update on new technical features and the most promising applications in cardiovascular diseases.

## Technical aspects

### General considerations

Intrinsic technical aspects of MRI technology such as high magnetic fields, quickly switching gradient fields, and radiofrequency signals may disturb photomultiplier tubes and electronics in “conventional” PET or PET/CT scanners. On the other hand, PET detectors in the field of view of MR scanners may deteriorate MR image quality by electromagnetic interferences and inhomogeneities. These issues were the reason why the construction of an integrated PET/MRI was a technically and also financially demanding project of long duration.

Two different constructions of an integrated whole-body PET/MRI are available, the first being a PET/CT connected with a stand-alone MR scanner connected via table (Philips Ingenuity TF PET/MR, GE-PET/CT-MR) (Zaidi et al. 2011). The second approach represents a truly integrated PET/MR system, where a PET detector ring is installed within the MR tube (Siemens Biograph mMR (Delso et al. 2011), GE SIGNA PET/MR (Levin et al. 2016)). The first construction approach circumvents the aforementioned issues of mutual interferences between PET and MRI. For the truly integrated PET/MRI scanners, novel detector technology had to be engineered: Siemens uses so-called avalanche photodiodes (APDs) (i.e., photodetectors made of lutetium oxyorthosilicate crystal which are insensitive to magnetic fields even at high field strengths) (Pichler et al. 2006), whereas in GE’s scanner silicon photomultiplier technology (SiPM)-based PET detectors are installed. One advantage of SiPM is the possibility of time-of-flight PET imaging with a temporal resolution of < 400 ps (Levin et al. 2014). Only these two vendors have truly integrated PET/MRI scanners on the market. Until today, around 100 Siemens Biograph mMR and 70 GE Signa PET/MRIs have been installed worldwide.

A problem which both integrated scanners had to address was the attenuation correction (AC). AC is obligatory for PET imaging in general and particularly for cardiovascular PET. The reasons for this need are that non-AC PET does not allow the assessment of absolute activity values and artifacts may severely hamper PET data, both of which hinder reliable interpretation of PET images.

### AC in PET/MRI

In stand-alone PET, AC was performed by rotating rod sources (e.g., Germanium-68), a very time-consuming matter. In PET/CT, CT data are used to correct for attenuation by making use of the fact that the density of tissue (and thus the attenuation coefficient) can be easily estimated via Hounsfield units. For integrated PET/MRI, different approaches had to be engineered, as there is no MRI sequence that allows to directly estimate the density of the imaged tissue.

### Ways to estimate AC-maps in integrated PET/MRI

The most frequently used approaches to generate AC-maps from MR data rest on templates, segmentation or atlases, and on PET emission data. Briefly, in the segmentation-based algorithm, the AC-map is generated by different tissue classes, each with a certain attenuation coefficient for 511 keV photons (Huang et al. 1981). This method is applied in the Siemens Biograph mMR where water and fat images are generated from a Dixon VIBE MR sequence to subsequently define air, lung, fat, and soft tissue in the AC-map (Martinez-Moller et al. 2009; Coombs et al. 1997). This method requires a breath-hold of about 18 s per bed position, which may cause problems in very sick patients. While there is an alternative approach using a T1-weighted (T1w) turbo spin echo sequence to shorten image acquisition time for AC-map generation, this approach neglects the density differences between fat and soft tissue (0.086 cm^−1^ vs. 0.096 cm^−1^) (Schulz et al. 2011). Recently, a novel approach to also model bone using an atlas was suggested and has now been implemented into the Siemens mMR (Paulus et al. 2015). The Philips Ingenuity TF utilizes T1w-GE MR images to segment the three different tissue classes air, lung, and soft tissue. In the GE Signa PET/MRI, a multi-station, whole-body, three-dimensional, dual-echo, RF-spoiled gradient recalled echo (SPGR) sequence (LAVA-Flex) is used and images are segmented into air, lung, fat, and soft tissue. While this approach is similar to that of the Siemens Biograph mMR approach, the difference lies in a classification variation for fat and soft tissue (Beyer et al. 2016). The restriction to a certain number of tissue classes may cause variations in the estimations of radiotracer uptake in thoracic tumors in the range of 5–23% (Martinez-Moller et al. 2009; Schulz et al. 2011; Samarin et al. 2012). For cardiac imaging, however, studies indicate very close agreement between PET/CT and PET/MRI for fludeoxyglucose (FDG) viability imaging (Lau et al. 2017).

**Fig. 1:**
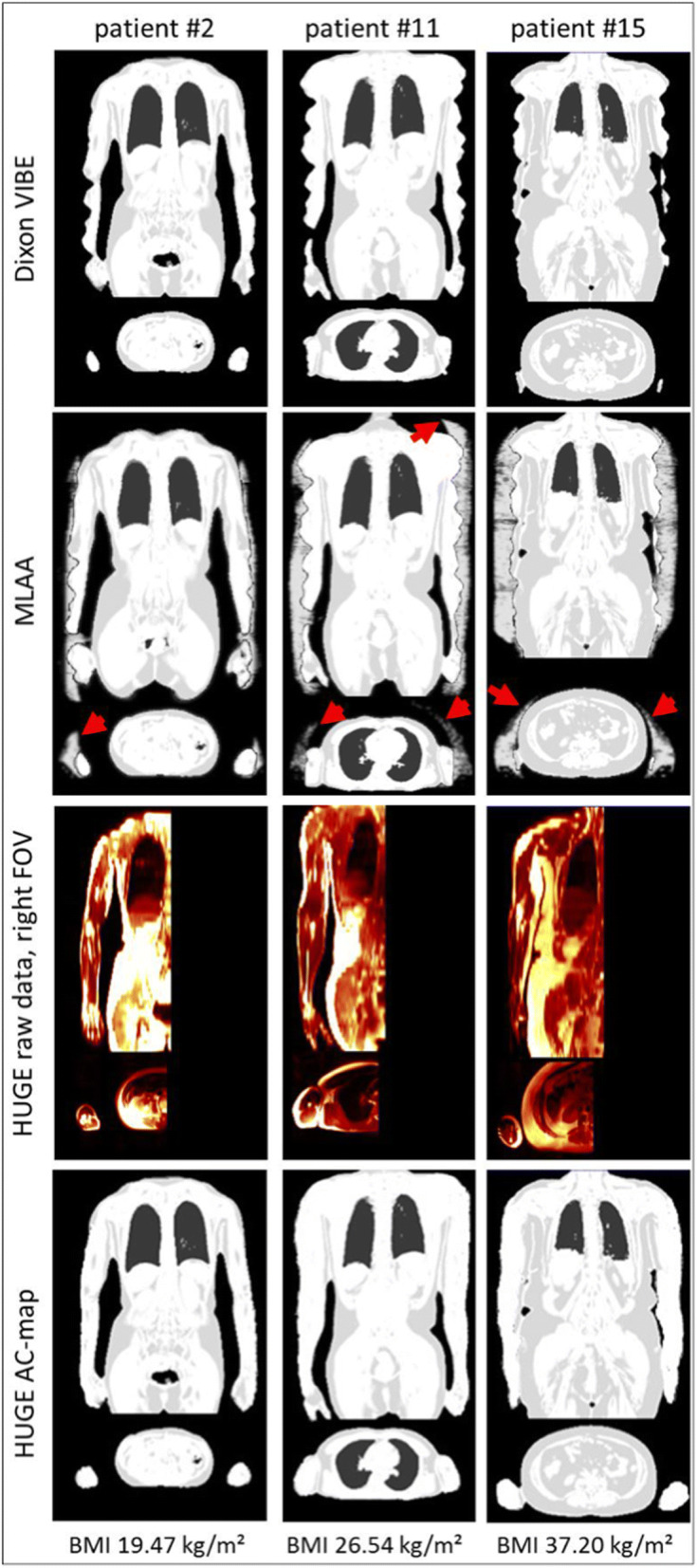
Attenuation correction (AC) map using different methods. AC-maps are shown for the Dixon-VIBE approach (upper row), complemented by MLAA (second row) or HUGE (third row) to recover truncated parts of the body. Red arrows indicate the overestimation around arms and shoulders when applying the MLAA algorithm. Examples of three different patients with increasing body mass index (from left to right) are shown. (Reprinted with permission from Lindemann, M. E., Oehmigen, M., Blumhagen, J. O., Gratz, M. and Quick, H. H. (2017), MR-based truncation and attenuation correction in integrated PET/MR hybrid imaging using HUGE with continuous table motion. Med. Phys., 44: 4559-4572. doi:10.1002/mp.12449)

The last approach that will be briefly discussed here is the emission-based approach (maximum likelihood estimation of activity and attenuation (MLAA)), where the non-attenuation corrected PET data are utilized to calculate (missing parts of) the μ-map. A major limitation of this approach is photon scatter (Nuyts et al. 1999). This approach must be mentioned as it is used in PET/MRIs to recover parts of the body (particularly parts of shoulders and arms) which are not “seen” by the MR scanner, but are in the (as compared to the MRI) larger field-of-view of the PET scanner and, of course, contribute to attenuation (Nuyts et al. 2012). A limitation of this technique is that tracers that have no or minor uptake in the truncated parts of the body may also be “overlooked” by this method. In the current version of the Siemens Biograph mMR, a novel approach to overcome this limitation has been introduced—the so-called B_0_ homogenization using gradient enhancement (HUGE) technique (Blumhagen et al. 2014). Briefly, the readout gradient is optimized by local compensation of B_0_ inhomogeneities via gradient enhancement, resulting in a reduction of truncations outside the field-of-view of the MR scanner. First studies indicate that the HUGE method may be more accurate than the previously used MLAA methodology, particularly when using tracers other than FDG (e.g., I-124) (Fig. 1) (Lindemann et al. 2017). The remaining two AC estimation methods have been described elsewhere and—as they are not implemented in current hybrid PET/MR scanners—will not be discussed here further (Rischpler et al. 2013).

Recently, a novel sequence for AC (the so-called CAIPIRINHA (controlled aliasing in parallel imaging results in higher acceleration) accelerated Dixon 3D-VIBE sequence) with higher resolution but identical scan-time (19 s per bed position) has been introduced (Freitag et al. 2017). The potential of this novel high-resolution sequence is to omit other diagnostic sequences with longer acquisition times, thus allowing to shorten the overall scan-time significantly (which represents one of the major disadvantages of PET/MRI in comparison to PET/CT).

### Artifacts in AC-map generation by contrast media and implants

**Fig. 2:**
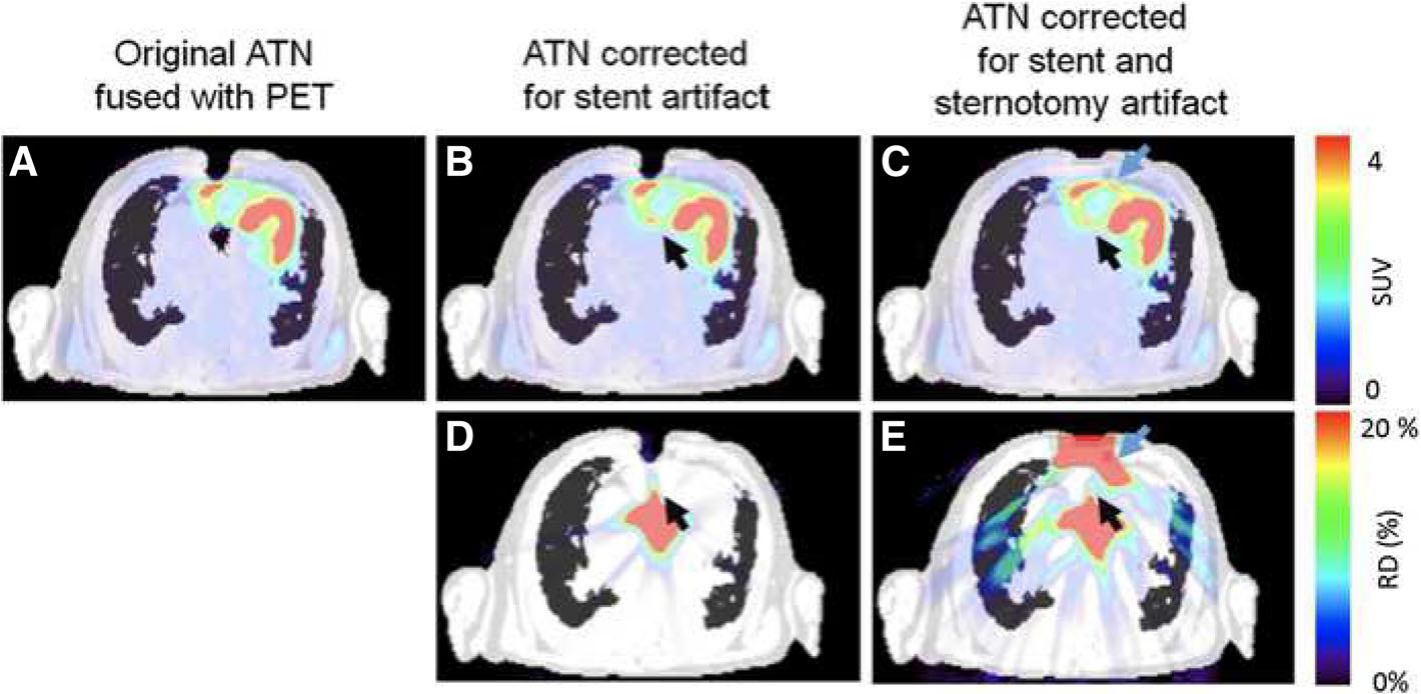
Artifacts in AC-map and AC-PET caused by foreign bodies. Foreign bodies such as sternal wires and artificial heart valves may cause artifacts in the AC-map, which translates into AC-PET (**a** overlay of original AC-map and resulting AC-PET). Corrections of the AC-map (**b** correction of susceptibility artifact by artificial aortic valve, **c** correction of both artifacts caused by artificial aortic valve and sternal wires) may have a significant effect of about 20% on subsequently calculated AC-PET (relative differences of the corrected AC-PET vs. non-corrected AC-PET, **d** artificial aortic valve, **e** artificial aortic valve and sternal wires). *AC* attenuation correction, *ATN* attenuation. (Reprinted with permission from Lassen, M.L., Rasul, S., Beitzke, D. et al. J. Nucl. Cardiol. (2017). 10.1007/s12350-017-1118-2)

In PET/CT, implanted devices (such as stents, sternal wires, defibrillators, pacemakers, cardiac resynchronization therapy devices) cause artifacts in CT which are translated into the AC-map and ultimately into the attenuation-corrected PET data, where regularly an overestimation of radiotracer uptake is observed. To consider for this issue, non-AC PET data are taken into account, particularly in FDG-PET/CT with the clinical question for infection of, e.g., prosthetic valves to avoid false-positive readings (Jimenez-Ballve et al. 2016). In PET/MRI, however, cardiac (non-magnetic metallic) devices cause a signal that exceeds the actual size of the causing foreign body, resulting in an underestimation of the attenuation and ultimately underestimation of tracer uptake. Obviously, all cardiac devices must be checked with caution if they are approved for a 3T MRI as the magnetic field may cause malfunction or heating (a detailed databank, where most devices’ compatibility with MRI may be checked can be found at www.mrisafety.com). However, as many novel cardiac devices enter the market (e.g., left atrial appendage closure devices, transcatheter aortic heart valves, pacing systems, event-recorder), it is often not clear whether or not a device is compatible with MRI and whether or not these devices cause artifacts which translate into PET data and may cause false readings. In a recent study of 20 patients with viability imaging by FDG and ammonia PET/MRI, different AC artifacts were analyzed (Lassen et al. 2017). The authors found artifacts caused by sternal wires or metallic implants in 20% and 30% of cases, respectively. Maximum relative differences in the SUVmean ranged from 11 to 196% in FDG or N-13 PET AC images when correcting for these artifacts (see Fig. 2).

**Fig. 3:**
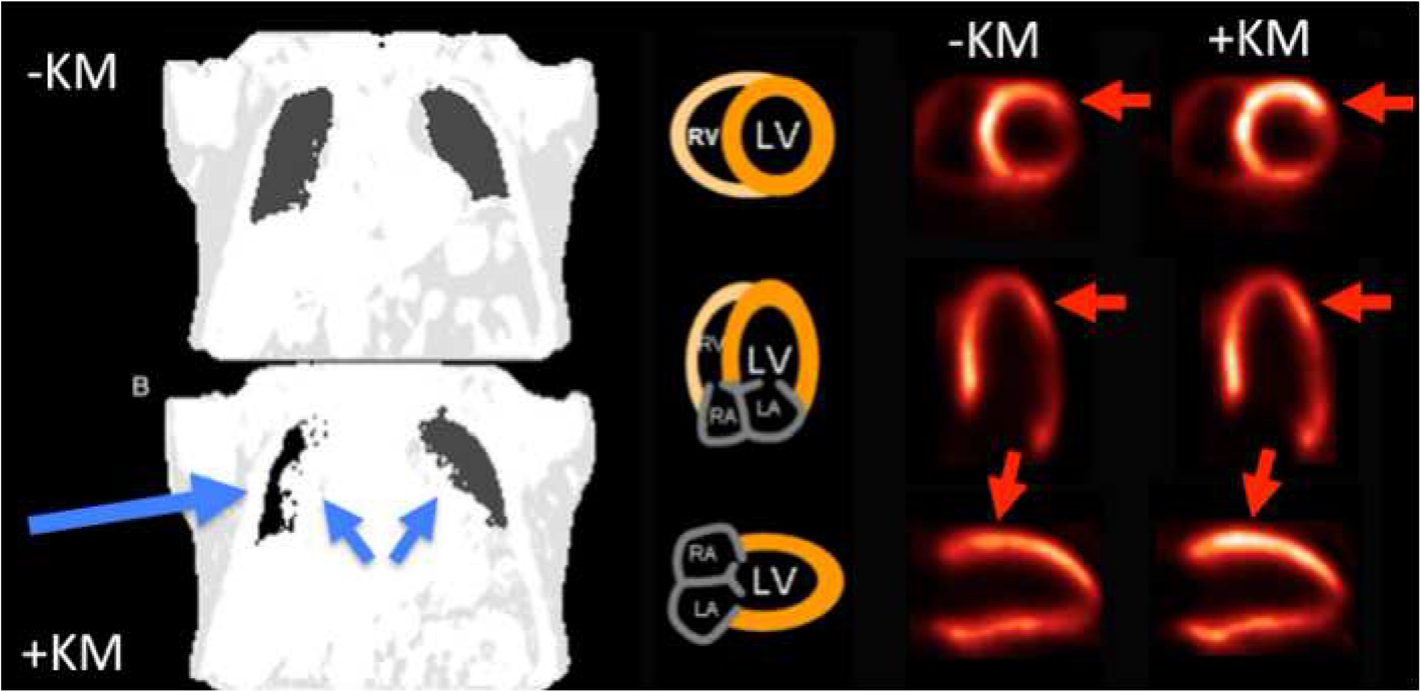
Effects of contrast media on attenuation-correction in PET/MRI. Attenuation correction (AC)-maps derived from Dixon VIBE MR sequences before (upper row, left) and after contrast media application (bottom row, left). Contrast media application may cause erroneous segmentation of the lungs (right lung: air instead of lung tissue indicated by large blue arrow) and in hilar regions (overestimation of soft tissues bihilarly, small blue arrows). This translates into an erroneous tracer uptake increase on attenuation corrected PET images in the anterolateral wall (red arrows). (Reprinted with permission from Rischpler C, Nekolla SG, PET Clin 11 (2016) 465–477 10.1016/j.cpet.2016.05.006)

Errors in segmentation are also seen when AC-maps are generated after the application of MR contrast media (Ruhlmann et al. 2016). MR contrast media reduces T_1_ values which translate into the Dixon VIBE sequence and may cause AC-map errors (Fürst et al. 2012) (see Fig. 3).

### Software solutions for image analysis

There are quite a number of free and commercial software solutions that offer detailed analysis of cardiac PET and MR imaging data. These products often work (semi-)automatically with only limited user interaction. Software applications that truly handle integrated quantitative image analysis of PET and MRI data are still scarce yet, and most software still are only able to handle modality and therefore cannot fully serve the temporal and spatial integrity of simultaneously acquired data. Furthermore, most software focus on oncologic applications and are not specifically designed for the analysis of cardiac imaging data, including the estimation of functional parameters (e.g., end-diastolic and end-systolic volume, ejection fraction), myocardial blood flow, or the generation or polar maps. There are a handful of software packages (e.g., OsiriX, Pixmeo SARL, Geneva, Switzerland; syngo.via, Siemens Healthineers, Erlangen, Germany) that allow fusion and analysis of fused PET and cardiac MRI data. Outside the scope of academic software developments, to the best of our knowledge, no software packages for fully integrated PET/MRI data—also quantitative in nature—is available. An approach for integrated analysis of cardiac PET and MRI data by co-registered polar maps and mutual segmentation of myocardial boundaries has recently been proposed and may allow for robust segmentation and immanent co-registration (Tezgah et al. 2017). Besides the analysis of simultaneously acquired PET/MRI data, most of the mentioned software packages also allow to fuse data from separately acquired PET(/CT) and MRI data. However, this often requires a high amount of user interaction and may result in misregistration. This may particularly be a problem in case of radiotracers that result in weak signals or bind only to a certain part of the heart (e.g., focal inflammation of the myocardium in cardiac sarcoidosis). In these cases, the user has to rely on the (unspecific) uptake of other surrounding structures such as the liver or bones. Another issue is that MRI data is often non-isotropic and that non-axial slices of the body (e.g., short-axis and long-axis of the heart) are acquired. Therefore, volumetric PET data has to be resampled to the respective MRI data. This may be particularly difficult if for example only a certain number of short axis slices were acquired on MRI (e.g., myocardial perfusion with MRI).

## Clinical applications

### Myocardial perfusion imaging

**Fig. 4:**
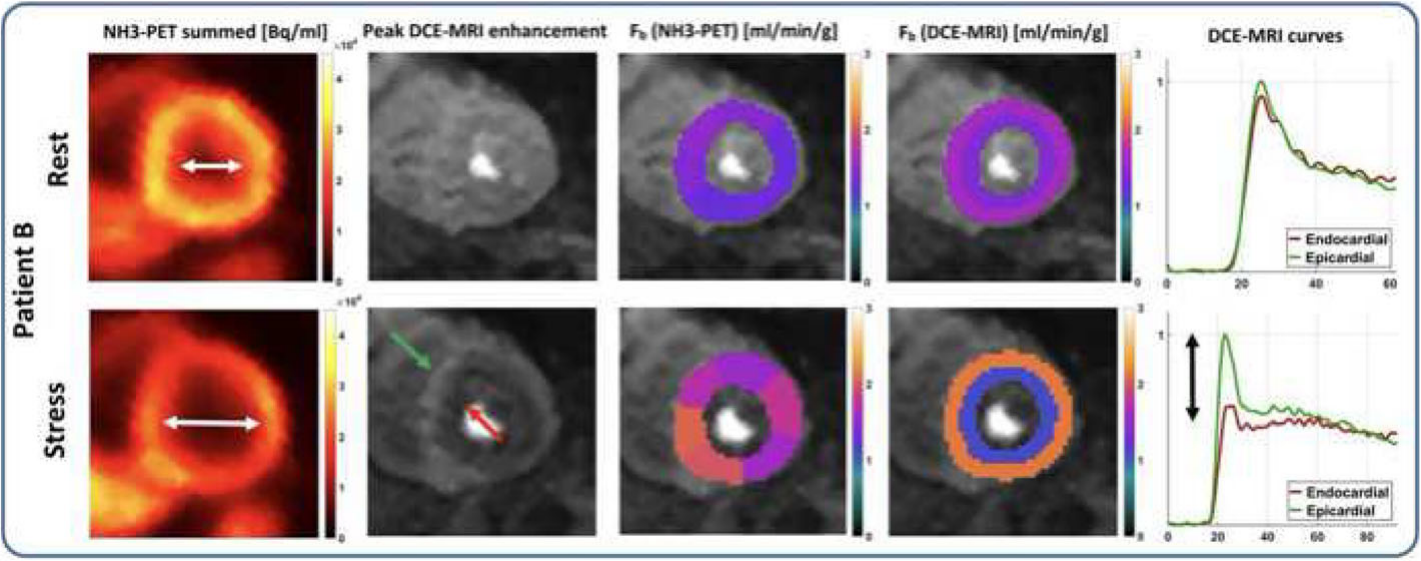
Hybrid PET/MRI myocardial perfusion assessment in a patient with hypertrophic cardiomyopathy. The apparent dilatation of left ventricular cavity during adenosine challenge (first column) is caused by a stress-induced subendocardial hypoperfusion as demonstrated by MRI MPI (column two to five). (Reprinted with permission from Kunze KP, Nekolla SG, Rischpler C, et al. Myocardial perfusion quantification using simultaneously acquired 13NH3-ammonia PET and dynamic contrast-enhanced MRI in patients at rest and stress. Magn Reson Med. 2018;00:1–14. 10.1002/mrm.27213)

Myocardial perfusion imaging (MPI) using PET or MRI are well-established procedures for the diagnostic work-up of coronary artery disease. PET MPI may be seen as the reference standard for quantitative assessment of myocardial blood flow and coronary flow reserve. Use of the superior characteristics of radiotracers (versus Gd-based contrast agents) and volume coverage of the whole left ventricle, MRI MPI has the advantage that it is performed without any ionizing radiation and has a higher spatial resolution so that even small areas of ischemic or scarred myocardium are detected. Therefore, hybrid PET/MRI may be useful to cross-validate the two techniques with respect to the complex procedure of quantification in MRI. This approach has just been published by Kunze KP et al. (2018). While a good correlation both on slice average and on a segment basis were found, MRI underestimated coronary flow reserve because of an overestimation of resting perfusion, regardless of the applied MRI deconvolution method. One possible synergistic aspect of PET and MRI was also demonstrated in this publication, i.e., the capability of MRI to distinguish subepicardial from subendocardial blood flow. The authors showed one patient with hypertrophic cardiomyopathy, who had an apparent cavity dilation on PET MPI which was caused by subendocardial ischemia as demonstrated by MRI MPI (Fig. 4). One disadvantage is that MRI MPI usually does not cover the whole left ventricle. As the extent of whole left ventricular ischemia is a key factor for therapy guidance (Hachamovitch et al. 2003), the estimation of this parameter may be inaccurate when using only three slices of the left ventricle. While approaches have been undertaken to enable 3D first-pass perfusion MRI for the estimation of myocardial blood flow in the whole left ventricle, this technique is far from being used in clinical routine (Wissmann et al. 2017). Another unfavorable aspect of MRI MPI is the use of Gd-chelate-based contrast agents. These extracellular contrast agents are not taken up by the viable cardiomyocytes and suffer from a low “extraction fraction,” which is also not only perfusion-dependent. These factors make quantitative myocardial perfusion assessment using MRI difficult. In contrast, radiotracers with short half-lives are used in PET MPI (the most commonly being O-15 water (*T*_1/2_ = 122 s), Rubidium-82 (*T*_1/2_ = 76 s), and N-13 ammonia (*T*_1/2_: 10 min)). While absolute blood flow quantification is routinely performed with these tracers in clinical routine in specialized centers, they have the disadvantage that an on-site cyclotron is a prerequisite or that a cost-intensive generator has to be purchased every month. These factors have prevented PET MPI to be a widely available technique. While novel ^18^F-labeled perfusion radiotracers are being developed—F-18 Flurpiridaz is the most prominent one—these radiotracers are still evaluated in clinical phase 3 trials (Rischpler et al. 2012; Berman et al. 2013). However, these radiotracers may make PET MPI with absolute flow quantification a more widely available method.

In the future, we expect that hybrid PET/MRI will remain an attractive tool for cross-validation of novel myocardial perfusion approaches, including novel sequences, novel quantification algorithms, novel contrast agents, and/or novel PET radiotracers.

### Imaging of myocardial viability

**Fig. 5:**
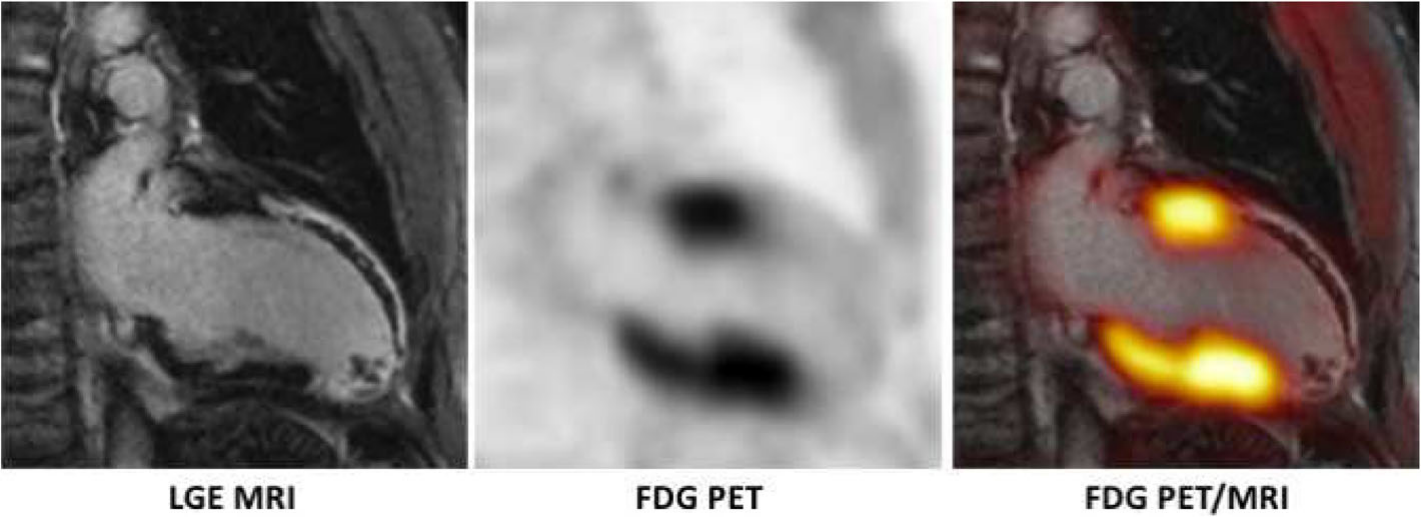
Viability assessment with hybrid FDG PET/MRI. A FDG PET/MRI (viability protocol with glucose loading) was performed in a mid-aged man early after acute ST-elevation myocardial infarction with occlusion of the left anterior descending (LAD) coronary artery. The inferior wall and the basal part of the anterior wall demonstrate no late gadolinium enhancement (LGE) and a normal FDG uptake and are therefore PET/MRI vital. The remaining parts of the heart in this two-chamber-view demonstrate transmural LGE with subendocardial microvascular obstruction in the anterior wall and almost absent FDG uptake and are therefore judged PET/MRI non-vital

Repetitive or chronic ischemia (i.e., continuous hypoperfusion) may cause myocardial wall motion abnormalities and a reduced left ventricular pump function. In such a condition, it is known that cardiomyocytes primarily metabolize glucose instead of free fatty acids and that viability of cardiomyocytes is maintained by a protective gene and protein program. This state of contractile dysfunction, however, may be reversible when normal perfusion is reestablished (Rahimtoola 1985) and is therefore often referred to as “hibernating myocardium” (Heusch 1998; Heusch et al. 2005). It is a well-known fact that the presence of hibernating myocardium is associated with poor outcome if not revascularized (Beanlands et al. 1998) and that the extent of hibernating myocardium may serve as an important marker for therapy guidance (Di Carli et al. 1994; D'Egidio et al. 2009; Allman et al. 2002). There are several methods to non-invasively assess myocardial viability (e.g., dobutamine stress echocardiography and MRI, Thallium-201 SPECT), with F-18 FDG PET and late gadolinium enhancement (LGE) MRI most often used (Tillisch et al. 1986). For FDG PET, the patient’s heart must be prepared in a way that glucose is the primary substrate and FDG uptake is thus increased. There are several methods to increase the expression of glucose transporters on the cell membrane of cardiomyocytes with oral glucose loading, and the hyperinsulinemic-euglycemic clamp technique is most often used. It is recommended to combine FDG PET with a perfusion study (e.g., N-13 ammonia PET or Tc-99 m sestamibi SPECT) in order to identify hibernating myocardium (mismatch between decreased perfusion and increased glucose consumption). Based on a meta-analysis, FDG PET has a 92% sensitivity among viability imaging approaches and an acceptable specificity of 63% (Schinkel et al. 2007). LGE MRI is often used as an alternative for viability imaging, even though the term viability is entirely misleading since scarred tissue is actually imaged. The theory behind this approach is that dysfunctional myocardium is more likely to recover the higher the subendocardial proportion of non-scarred (= viable) myocardium. The technique makes use of the fact that Gd-chelate-based contrast agents have a reduced wash-out from areas with increased extravascular space (such as the fibrotic tissue in a myocardial scar) in comparison to a fast wash-out from “healthy” non-scarred myocardium (Klein et al. 2004; Klein et al. 2007). Even though the approaches are quite different (molecular imaging of an increased glucose consumption in viable cells vs. extracellular contrast agent accumulation in fibrotic tissue), a good agreement regarding myocardial viability assessment has been demonstrated (Klein et al. 2002). Benefits of LGE MRI over FDG PET include the lack of ionizing radiation, the highly reproducible accumulation of Gd-based contrast agents in scar tissue, independently from the metabolic state of the patient (which may be an important factor in certain patients such as diabetics), and the high in-plane resolution of MRI of about 1–3 mm, which may permit the distinction between thinned myocardium and scarring (e.g., in the case of a reduced FDG uptake) or the depiction of tiny areas of myocardial scar, which are also known to carry prognostic significance even in patients without overt myocardial infarction (Kim et al. 2000; Kwong et al. 2006). Studies on viability imaging using hybrid FDG PET/MRI are still scarce. In the three available studies, agreements between FDG PET and MRI were reported as moderate to substantial (Fig. 5) (Priamo et al. 2017; Rischpler et al. 2015; Nensa et al. 2013). In one study with a very small patient cohort, adding FDG PET to LGE MRI resulted in a reclassification of about 19% of segments (Priamo et al. 2017). However, no follow-up to validate the actual impact in terms of wall motion recovery was available.

While this latter study was performed prior to elective revascularization for coronary artery disease, the other two studies were performed early after myocardial infarction. In one study, hybrid FDG PET/MRI was performed early after myocardial infarction in order to compare LGE MRI and FDG PET in dysfunctional myocardium (Rischpler et al. 2015). The aim was to investigate the functional recovery of the dysfunctional segments after 6 months. A high intermethod agreement for the transmurality of LGE and the reduced FDG uptake in the affected segments was found (*Κ* = 0.65). The wall motion of both the “PET viable” (segments without or only mild decrease in FDG uptake) and the “MRI viable” (non-transmural LGE < 50% of the myocardial wall) dysfunctional segments was more likely to recover after 6 months. A novel finding was segments which demonstrated a “discordant” pattern of LGE transmurality and FDG uptake, i.e., a reduced FDG uptake but non-transmural or even only minor LGE. Importantly, only around 40% of these “discordant” segments demonstrated an improvement in wall motion after 6 months. In a study by Nensa et al., the alteration of glucose metabolism in the area of infarction was set in relation to the actual infarct size (assessed by LGE MRI) and to the area at risk (determined by the so-called ESA (endocardial surface area) method) (Nensa et al. 2015). The ESA method defines all myocardial segments as area at risk that demonstrate at least some degree of LGE regardless of the transmurality of LGE. As expected, it was found that the area at risk was larger than the infarct area (31 ± 11% vs 10 ± 10%). The most interesting finding was that the area at risk correlated with the extent of altered glucose metabolism indicating that post-ischemic myocardium demonstrates reduced FDG uptake. Still, the pathophysiological background of such metabolic alteration of the myocardium remains unclear and warrants to be further investigated. Until today, there is no study available that investigated the value of integrated viability FDG PET/MRI in patients prior to revascularization with a follow-up examination in order to estimate the predictive value of the combined approach in this setting.

### Imaging of focal inflammation

Using MRI, several processes which may reflect inflammatory processes can be detected. Among those are myocardial fibrosis (accessible using LGE or T1 mapping) and myocardial edema (T2-weighted sequences) (Heusch et al. 2015). Also, pericardial effusion and wall motion abnormalities can be detected with high sensitivity. However, in recent years, FDG PET is increasingly used for the assessment of inflammatory processes in the heart (Erba et al. 2013). FDG PET allows to detect and monitor the extension and grade of the respective inflammatory process which, in turn, allows to distinguish between florid and healed inflammatory processes. As described above, FDG may also be taken up by vital cardiomyocytes when the heart’s metabolism runs on carbohydrates. Consequently, an imperative prerequisite for FDG imaging of inflammation is a switch of myocardial metabolism toward the oxidation of free fatty acids. Common patient preparation protocols for this purpose include a high-fat low-carb diet on the day prior to the scan, a prolonged fasting period (e.g., > 12 h), and the injection of heparin before the administration of FDG (Nensa et al. 2017; Scholtens et al. 2016). With such protocol, glucose uptake in healthy, non-inflamed myocardium is decreased to a minimum level, and FDG uptake consequently reflects pathological processes, such inflammation. This approach may fail, particularly in non-compliant patients, in diabetics and in patients on certain medications such as glucocorticoids, emphasizing an unmet clinical need for PET radiotracers specifically targeting inflammatory processes. Some studies indicate that the chemokine receptor 4 (CXCR4), which may be targeted with high affinity and specificity using Ga-68 pentixafor, is a promising target (Rischpler et al. 2016a). However, CXCR4 is a very complex target which is highly variable and dynamic and is expressed on various cells, including leukocytes, sprouting vessels, and cancer cells. Also, F-18 NaF has been identified as a potential radiotracer to detect plaque vulnerability or to image scar tissue after myocardial infarction, even though the process of tracer accumulation is not completely understood (Marchesseau et al. 2018).

#### Myocarditis

**Fig. 6:**
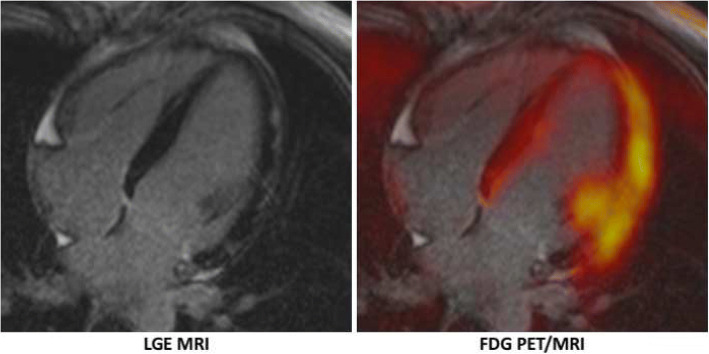
FDG PET/MRI in a case of myocarditis. Subepicardial, mostly band-shaped late gadolinium enhancement (LGE) on MRI in the lateral wall of the left ventricle. The FDG uptake on PET clearly exceeds LGE MRI indicating active infection exceeding the scarred areas

Myocarditis is an inflammatory disease of the heart that may be caused by various noxae including microbial infection, hypersensitivity reactions, or autoimmunological diseases. Still, the reference standard for the diagnosis is endomyocardial biopsy with the disadvantages of invasiveness and a high rate of complications. Another problem of endomyocardial biopsy is that sampling errors may occur, as specimens may be taken from not affected parts of the myocardium. This in turn may result in discrepancies between imaging and biopsy results. Consequently, the implementation rate of endomyocardial biopsy is variable between different centers. Another consequence is that non-invasive imaging, particularly MRI, has seen a great increase over the last years for this indication and that clinical decision and therapy is often guided by this imaging modality. The procedure of the MRI scan and its reporting has been standardized by the “Lake Louise Criteria.” The imaging protocol includes the minimum of hyperemia (early gadolinium enhancement), edema (T2-weighted imaging), and necrosis/scar (late gadolinium enhancement) imaging, resulting in rather sobering numbers for sensitivity, specificity, and negative predictive value of 67%, 91%, and 69%, respectively, when using endomyocardial biopsy as the gold standard (Friedrich et al. 2009). A drawback is that MRI primarily depicts morphological changes of tissue damage but the activity of the inflammatory processes is assessed only indirectly. The combination of PET with MRI may contribute to the solution of this problem. Following initial positive case reports on the use of hybrid FDG PET/MRI for diagnosing, grading, and monitoring myocarditis (Nensa et al. 2014; von Olshausen et al. 2014; Piriou et al. 2015), Nensa et al. published the first prospective study in 65 patients on this matter (Nensa et al. 2018b). In comparison to a variant of the “Lake Louise Criteria” as reference, FDG PET had a sensitivity of 74% and a specificity of 97%. Most importantly, one patient with biopsy-proven myocarditis had pathological myocardial FDG uptake in the absence of abnormalities in MRI, possibly representing an early stage of the disease, yet without morphological changes of the heart. Another recent publication, however with a very small number of patients, also demonstrated the feasibility of this approach (Hanneman et al. 2017). An open question remains, however, whether or not combined FDG PET/MRI results in an improved diagnosis and patient outcome. An example of a patient with myocarditis who was imaged on a hybrid PET/MRI is depicted in Fig. 6.

#### Cardiac sarcoidosis

Another inflammatory disease where hybrid PET/MRI may play a pivotal role is the cardiac involvement in sarcoidosis. In up to 40% of cases, the initial clinical manifestation of this insidious disease is sudden cardiac death (Sekiguchi et al. 1980). In case of cardiac involvement, the myocardium is infiltrated with inflammatory granulomas which may result in scarring, malignant arrhythmia, and heart failure. It is obvious that cardiac involvement in sarcoidosis must be ruled out reliably. Usually, the guidelines of the Japanese Ministry of Health and Welfare are used to estimate the probability of cardiac involvement. However, these guidelines only recommend cardiac MRI or Ga-67 scintigraphy as imaging tests and not FDG PET. Accumulating evidence suggests a valuable and important role of FDG PET regarding the diagnosis, monitoring, and therapy guidance of cardiac sarcoidosis and an expert consensus clearly recommended this method (Birnie et al. 2014; Slart et al. 2018). A myocardial perfusion study (such as N-13 ammonia PET) may add to the FDG PET for the differentiation between early stage, advanced stage, or healed cardiac sarcoidosis, and also have prognostic implications (Blankstein et al. 2014). In principle, FDG PET may be combined with MRI perfusion. MRI MPI, however, only images a certain number of slices (most often three). There are approaches to perform MRI-based MPI of the entire left ventricle, but this is not implemented in clinical routine yet (Wissmann et al. 2017). Therefore, certain parts of the heart (such as the apex) may not be adequately assessed with a MRI MPI/FDG PET approach. The combination of LGE MRI with FDG PET may have an additional benefit, in the exact illustration of myocardial scarring which is important in the case of ICD implantation (Schneider et al. 2014). While there are some case reports and smaller studies on hybrid FDG PET/MRI for cardiac sarcoidosis, a prospective trial with larger patient numbers is still missing (Hanneman et al. 2017; Schneider et al. 2014; Wada et al. 2016; Kiko et al. 2018; Wicks et al. 2018). An alternative approach may be the assessment of the inflammatory granulomas in the myocardium using radiotracers targeting the somatostatin receptor (e.g., Ga-68 DOTANOC). While first reports using this approach are available for PET/CT, there is no clinical experience with simultaneous PET/MRI (Slart et al. 2017; Gormsen et al. 2016).

#### Imaging post-ischemic inflammatory processes in the myocardium

**Fig. 7:**
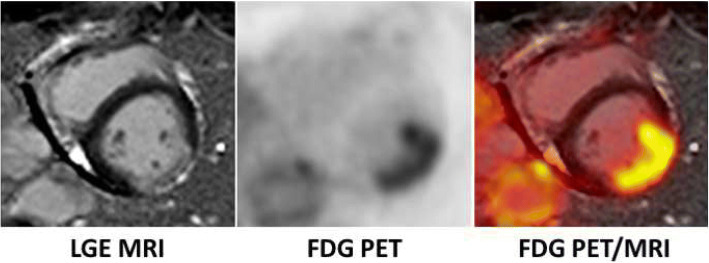
Hybrid ^18^F-FDG PET/MRI (suppressed myocardial glucose metabolism) early after ST-elevation myocardial infarction. LGE MRI (left panel) demonstrates myocardial infarction scar of the lateral wall. On FDG PET (middle panel), an intense FDG uptake matching the scar (right panel) is observed. The glucose metabolism in the scar indicates a florid inflammatory response after myocardial infarction

There is an intense inflammatory process in the myocardium after acute myocardial infarction which is orchestrated by different immune cells such as neutrophils and monocytes/macrophages (van der Laan et al. 2012). This inflammatory response is a key factor regarding the development of heart failure after myocardial infarction. In addition to established prognostic factors such as infarct size and ejection fraction, inflammation came into the focus of cardiovascular research in recent years (van der Laan et al. 2012; Curley et al. 2018; Heusch et al. 2014). Lee et al. recently demonstrated in a rodent model of myocardial infarction that the invasion of the myocardium by monocytes can be imaged using FDG PET/MRI (Lee et al. 2012). This imaging approach was subsequently translated into a clinical setting (Wollenweber et al. 2014), and a relation between the post-ischemic myocardial inflammation and the FDG uptake in spleen and bone marrow (as a marker of systemic inflammation) was suggested. Indeed, post-ischemic FDG uptake in the heart is a prognostic marker for left ventricular remodeling after myocardial infarction, independently of infarct size assessed by LGE MRI (Fig. 7) (Rischpler et al. 2016b). Consequently, FDG PET/MRI may be a very promising tool for risk stratification of patients after myocardial infarction regarding the development of heart failure, and it may serve to investigate effects of (novel) interventions with the aim to modulate this inflammatory response.

## Conclusion

Since our first review on cardiac PET/MRI in 2013 (at a time when not a single publication on integrated cardiovascular PET/MRI was available), users have gained more experience with this modality, and the number of potential applications has increased. For myocardial perfusion imaging, hybrid PET/MRI remains mainly a research tool for cross-validation of PET vs. MRI and for the development of new sequences, tracers, and contrast agents. For viability imaging, first publications indicate a possible synergistic effect of FDG PET and cardiac MRI. For inflammatory focus imaging, particularly sarcoidosis and myocarditis, accumulating evidence suggests a clear benefit of this modality. In fact, in the institutions of the review’s authors, myocarditis and sarcoidosis are primarily examined using FDG PET/MRI (with suppressed myocardial glucose metabolism). For research questions, PET/MRI has the advantage that biosignals (e.g., using novel tracers) can be derived from PET, while MRI provides information on left ventricular pump function and morphology of the heart. In a review from 2016 on the utilization of PET/MRI in 39 sites, most centers expected an increase in cardiovascular utilization. Also, in a recent workshop at the annual meeting of the Society of Nuclear Medicine and Molecular Imaging at Philadelphia (USA) in 2018 with nine experienced PET/MRI centers, most users expected a growing number of cardiovascular PET/MRI studies. In conclusion, cardiovascular PET/MRI has arrived in clinical routine and has gained appreciation in the cardiovascular community.

## Abbreviations

AC: Attenuation correction
APD: Avalanche photodiodes
CAIPIRINHA: Controlled aliasing in parallel imaging results in higher acceleration
CT: Computed tomography
CXCR4: Chemokine receptor 4
ESA: Endocardial surface area
FDG: Fludeoxyglucose
HUGE: B_0_ homogenization using gradient enhancement
LGE: Late gadolinium enhancement
MLAA: Maximum likelihood estimation of activity and attenuation
MPI: Myocardial perfusion imaging
MRI: Magnetic resonance imaging
PET: Positron emission tomography
SiPM: Silicon photomultiplier
SPECT: Single-photon emission computed tomography
SPGR: Spoiled gradient recalled echo
SUV: Standardized uptake value
